# Supplementary material for: Treatment completion of drug-resistant tuberculosis in Ethiopia: A perspective from healthcare users
Source: PLOS Glob Public Health. 2025 Feb 7;5(2):e0004110. doi: 10.1371/journal.pgph.0004110 (PMC11805356; doi:10.1371/journal.pgph.0004110)
Supplement: Attachment — Submitted filename: Response_to_reviewers_auresp_2.docx [file pgph.0004110.s003.docx]

Response to reviewers

Reviewer 1

1. The paper contains instances whereby the authors mention studies (plural) and only cites one source.

- Corrected

2. The authors did not identify the limitations of the study.

- Addressed.

3. The authors do not provide further information on the semi-structured interview, how did the authors develop the structure of the interviews? Is there information on this?

- Explained in the data collection and management section.

4. In the discussion section the authors link the results to previous research well, it might be considered to add a sentence or two on practical ways to address the barriers highlighted in the study.

1. Incorporated in the recommendation part.

5. The reference list contains up-to-date citations, however it is not clear to the reviewer if the formatting of the references are accurate.

6. In some instances the font styles and spacing are not uniform.

- Corrected.

Reviewer 2

2. The authors use MDR-TB, RR-TB and DR-TB interchangeably – each of these refer to a specific phenomenon. Please be mindful when describing and try to be consistent.

- Corrected.

Abstract

3. The authors would benefit from a language edit, simplifying some of the very lengthy/verbose sentences. E.g., the fourth sentence in the abstract stretches over four lines and can be shortened/rewritten. In addition, the authors often revert to passive voice.

- Corrected

4. In the abstract, the results listed are sparse and more details are needed. While the authors set out to describe the barriers to treatment completion, it would also be interesting to note facilitators, or ways that participants overcome some of these barriers.
5. In the abstract, the conclusion and recommendations does not seem to match the findings. The authors suggest that national-, policy-, and facility level interventions are needed, but some of the barriers identified are interpersonal. A bit more justification is needed – or an expanded description of the findings.

- A more elaboration is added in the conclusion part.

Introduction

1. The authors provide several statements without substantiation/references. E.g., paragraph 5, sentences 1 and 2 both need references. While the third sentence does include references, it is not clear what “a number of variables” refers to?

The fourth paragraph is repetitive and can be deleted.

- Addressed

Materials and Methods

1. Study setting
   Include some details on whether these settings are all urban/rural, formal/informal, high/low TB burden, high income/low-income settings.

- Addressed.

2. Population
   How were participants recruited?

- Addressed
- Purposive sampling was used to recruit study participants, with the head nurses assisting in the process.

3. Data collection and management
   Several typos in this section.
   Which if the authors were involved in data collection/analysis?

- the first author

4. Data analysis
   This section needs several references. Content analysis is a method to quantify qualitative data. If the researchers conducted content analysis, they would need to specify their coding frame used to identify quantifiable patters to base their conclusions on. From the description of the findings, it seems like they used thematic analysis – but they would need to revise their description of the analysis process and include relevant references.

- Reference added and coding frame uploaded separately.

5. Results

Most of the participants were male – is this reflective of the TB epidemic in Ethiopia?

- May be. According to WHO 2021 TB report, among notified TB cases case adult women were 39%, adult men were 51%, and children were 10%. It is now included in the introduction part.
- It is included in the introduction part as per comment given.

Marital status – was “co-habiting” or “in a relationship” offered as options?

- No. In Ethiopia these are not familiar within the culture.

The authors note that these are participants who were previously treated. Did all complete treatment or did some default on treatment? How many episodes have the previously had? If available, it would be worth including this information in the demographic section.

Throughout this section it would be helpful to see the age of the participants along with the gender when quotes are provided.

- They are either lost to follow up or failures with previous treatment.

Sub-theme 1.1
The participants mentioned that they thought they would not be able to adhere for an extended time because of the smell of the tablets. Did they adhere?

- it was mentioned as one of the barriers to adherence

Sub-theme 2.1. Consulting after a long illness
It is not clear how this theme presents a barrier to adherence. Do the authors suggest that illness severity/progression is a barrier to adherence? It also seems similar to theme 2.2. The quotations illustrate that TB is hard on the body but not how it relates to treatment adherence.

- Consulting after a long illness when the disease becomes serious, causing harm to their lungs, might hinder successful treatment completion and cure. It is not about adherence.

Sub-theme 5.3. Accessibility

This section seems to be specifically related to rural/urban access to healthcare. Please indicate how many of the participants are from rural areas in your demographic section and relate it to the quotes provided in this section.

- DR-TB treatment centers are located in few zonal towns in the country and both rural and urban patients are affected by distance. Both are exposed for house rentals, transport costs, leaving work …

- Sub-theme 6.2. Returning to habits already quitted The quotes provided are for perceptions of other patients. Did any participants report that they reverted to these habits?

- Yes, they are sharing their lived experiences.

- Sub-theme 7.1. Lack of responsiveness
This subtheme does highlight an important concern related to quality of care. However, it does not illustrate challenges with adherence (when hospitalised, would patients not be more likely to take/complete treatment – even if administered by rude staff or without adequate explanation)?

- It affects successful treatment completion.

Discussion

Second paragraph: Please describe where these studies were conducted and when. There have been some improvements in available treatment for people diagnosed with MDR-TB. If these studies are older, the adverse events might have been more severe.

- 2019, 2020 Tanzania, India, and metanalysis of many studies

Third paragraph: “In line with this, the in-depth interview with health care providers indicated that DR-TB patients are not usually diagnosed timeously and come after multiple first line treatment courses” I tis unclear if this sentence (a) refers to a previous study in the literature, or (b) if the authors also conducted interviews. If (a) please make clear, if (b) it will need to be included in the methods, findings, etc.

- Thank you for the comment. The sentence is removed.

The authors describe how side effects such as vomiting hinders adherence. However, it is not clear how sub-optimal dosing is a barrier to treatment adherence. While this is a clinical concern, it is a consequence rather than a barrier.

- Sub-optimal dose is a barrier to successful treatment completion. Because it results in unfavourable treatment outcomes (failure, death) and hinders treatment success.

Conclusion

- Recommendations are added to address all levels in the health system.

“.. the researcher started each discussion sessions

- Corrected

“…to ensure that the researcher captures what they accurately.”

- Corrected

“Informed consent form provided and permission sought from the participants.”

- Corrected

ADR

- Addressed

Reviewer 3

Minor Comments:
1. The abstract - is unbalanced by sub-section, more must be presented under 'results' and less under background and conclusion sub-sections. The results that are presented are only descriptive, and do not help the reader actually understand what you found. Examples from the categories, not just broad themes are necessary.

- Addressed

2. The first three sentences of the background are without citations. There are readily available reports on drug resistant TB epidemiology that should be cited to establish the magnitude of the issue.

- I left to put citations in the abstract. But all of the sentences were cited in the introduction section with proper citations.

3. The third and fourth paragraphs of the introduction are unnecessarily repetitive.
4. Top of page 7 - there is more than anecdotal evidence that recurrence is more likely than first-time disease episodes. See literature on post-TB lung disease.
5. Top of page 8, first two sentences are unnecessarily repetitive.
6. Under section 2.4, we do not need to read again which hospitals were part of the study.

- Hospital names removed

7. Under section 2.5, please provide citations for 'a conventional content analysis'.

- Addressed [15]

8. Under section 2.5, "ATLAS.ti", not "Atlas. ti"

- Corrected

9. First paragraph under section 3 - are the participants typical of people with DR / RR-TB locally?

- DR TB include all forms of drug resistant TB including RR, MDR, Pre-XDR, XDR.

10. Top of page 25 - what reagents were needed / provided?

- Corrected

11. By way of example, in sub-section 2.2, we are twice told four hospitals (each named / listed) are where the data were collected. This could be shortened by writing something like: "The study settings were four DR-TB treatment-initiating hospitals in Ethiopia. Saint Peter TB Specialised and Alert hospitals are located in Addis Ababa, while Adama Referral and Bishoftu General Hospitals are located in Oromia Region in Adama and Bishoftu towns, respectively."

- Corrected by changing the sentecnce as in the above example.

12. But then this is further compounded when the very next sentence (sub-section 2.3) reads "Previously-treated DR-TB patients at Bishoftu, Saint Peter, Alert and Adama hospitals, who were willing to provide informed consent to participate in the study and who were 18 years and above were included in the study." - why would we need the four hospitals listed a third time in three sentences?

- Corrected by removing the names of the hospitals

13. And then even this is compounded even further when information from that sentences (participants had to be >17-years-old) is repeated again by presenting the logical extrapolation that people "less than 18 years were excluded from the study".

- Corrected by removing the sentence.

14. And as a final compounding of the problem, the paragraph ends with a sentence new to the revision of "Purposive sampling was used to recruit study participants.", but this not actually address the point raised by the reviewer that we need to know why these hospitals were selected, how patients treated at those hospitals were selected (i.e., for what purpose was the purposive sampling), nor the actual recruitment process (i.e., did you phone patients from information recorded on a register or something like that? If so, how many did not answer / declined participation etc.).

- Addressed by incorporating the following
- Hospitals were selected based on their DR-TB treatment service duration and patient numbers.
- Purposive sampling was used to recruit study participants, with the head nurses assisting in the process.
